# Digestive properties in fermentation driven interactions of plant polysaccharides with gut microbiota and their health implications

**DOI:** 10.3389/fphar.2026.1733849

**Published:** 2026-05-07

**Authors:** Ziye Jiang, Bo Li, Ran Zhao, Xuyan Zhao, Xiaoyu Zhang, Lili Jiao

**Affiliations:** 1 Jilin Ginseng Academy, Changchun University of Chinese Medicine, Changchun, China; 2 School of Pharmaceutical Sciences, Changchun University of Chinese Medicine, Changchun, China

**Keywords:** digestive properties, fermentation, gut microbiota, plant polysaccharides, prebiotics, short-chain fatty acids

## Abstract

Plant polysaccharides, as pivotal dietary metabolites, primarily exert their health-promoting effects through interactions with the gut microbiota. These carbohydrates are resistant to digestion in the upper gastrointestinal tract and can reach the colon intact, where they are fermented by the gut microbiota to produce a variety of bioactive metabolites. Notably, the bioactivities of plant polysaccharides rely on microbial fermentation rather than direct absorption by the host. Gut microorganisms degrade diverse plant polysaccharides using carbohydrate-active enzymes (CAZymes) encoded by polysaccharide utilization loci (PULs) and via cross-feeding mechanisms.This paper systematically reviews the fermentation-driven dynamic interactions between plant polysaccharides and the gut microbiota, focusing on five core aspects: (1) The digestive resistance of plant polysaccharides as a prerequisite for colonic fermentation; (2) Microbial degradation mechanisms of plant polysaccharides; (3) Changes in the physicochemical properties and structure of plant polysaccharides during fermentation and their effects on bioactivity; (4) Selective proliferation of beneficial bacteria and regulation of microbial metabolism by specific polysaccharides acting as prebiotics; (5) Health benefits of plant polysaccharides mediated by the gut microbiota. By integrating recent advances in the digestibility of plant polysaccharides and microbial fermentation mechanisms, this paper comprehensively elucidates the complex regulatory network underlying the bioactivities of plant polysaccharides, and provides a scientific basis for the targeted application of these natural products in promoting human health and preventing diseases.

## 1 Introduction

**FIGURE 1:**
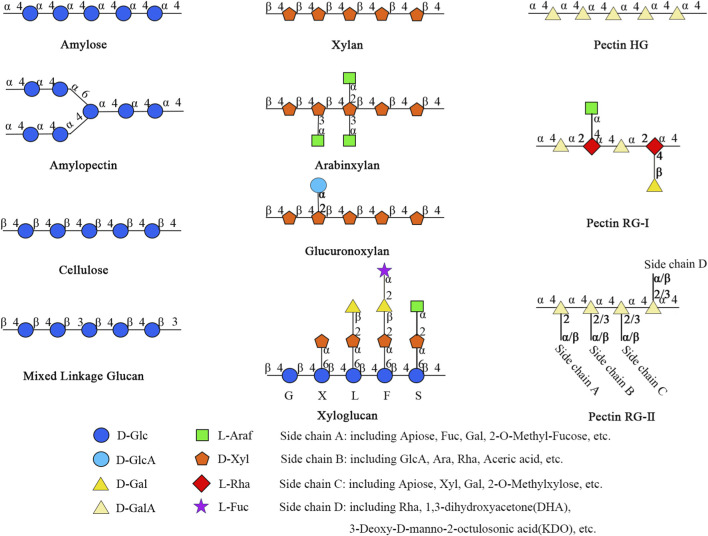
Examples of typical plant polysaccharide structure.

Dietary metabolites have a profound impact on human health, with plant-based foods playing a central role. Among its key metabolites, plant polysaccharide is a complex carbohydrate composed of more than ten monosaccharide units connected by glycosidic bonds, which is particularly important (Chen et al., 2024a; Li et al., 2024b; Lin et al., 2024). These natural polymers originate from various plant resources, including fruits (such as red dates and figs), stems/leaves (such as mulberry leaves and aloe vera), and roots/stems (such as polygonatum and kudzu root) (Chen et al., 2024a; Li et al., 2024b; Lin et al., 2024), exhibiting remarkable structural diversity (Figure 1). Functionally, they encompass structural metabolites (cellulose, hemicellulose) and energy-storing molecules (starch), as well as metabolites with unique physiological activities (such as astragalus, ginseng, and wolfberry polysaccharides). These active metabolites are renowned for their immune-regulating, antioxidant, and hypoglycemic effects (Li et al., 2022b; Zhou et al., 2023).

The gut microbiota, often referred to as the “second genome” (Li et al., 2020a), constitutes a vast and intricate microbial ecosystem in the human intestine. This biological community plays a vital role in host health by regulating metabolic processes, immune development, and pathogen resistance (van der Hee and Wells, 2021; Sinha et al., 2022), while also serving as the primary force in digesting plant polysaccharides. Most plant polysaccharides (particularly non-starch types) resist enzymatic degradation in the upper digestive tract (oral cavity, stomach, small intestine) due to mammals’ lack of specific endogenous CAZymes (Chen et al., 2018a; Ge et al., 2021; Wardman et al., 2022). This digestive resistance ensures that they reach the colon largely intact. Here, they become the main substrate for microbial fermentation, and this process is the fundamental basis of their bioactivity (Ge et al., 2024; Xue et al., 2025). The gut microbiota possesses abundant CAZymes (Kaoutari et al., 2013) that break down complex polysaccharide structures into simple sugars. These sugars are then fermented into various metabolites, most notably short-chain fatty acids (SCFAs) like acetic acid, propionic acid, and butyric acid, along with other metabolites such as succinic acid, lactic acid, and gas (van der Hee and Wells, 2021; Zhang et al., 2022b). These fermentation products are not mere waste; they serve as energy sources for colonic cells, act as signalling molecules influencing host metabolism and immunity, and shape the microbial environment itself (Topping and Clifton, 2001; van der Hee and Wells, 2021; Chen et al., 2024c). Additionally, plant polysaccharides function as prebiotics, selectively stimulating beneficial bacteria through fermentation to enhance their growth and activity while modulating the overall structure and functionality of the intestinal microbiota (Zhang et al., 2022c).

The interaction between dietary plant polysaccharides and gut microbiota forms the cornerstone of nutritional science and host-microbe symbiosis. Understanding the complexity of this fermentation-driven interaction is crucial. The remarkable structural diversity of plant polysaccharides including variations in monosaccharide composition, glycosidic bond types, branching patterns, molecular weight, and modifications, determines their susceptibility to microbial enzymes, which in turn dictates specific fermentation characteristics and downstream physiological effects (Li et al., 2024b; Yao et al., 2024). In turn, microbes have evolved sophisticated polysaccharide utilization strategies, including specialized enzyme systems typically encoded in polysaccharide utilization sites (PULs) (particularly prevalent in Bacteroidetes) and cross-feeding metabolic strategies in which metabolites produced by primary degraders are utilized by other microbial communities (Kaoutari et al., 2013; Heintz-Buschart and Wilmes, 2018; Fernandez-Julia et al., 2021; Beidler et al., 2023). While significant progress has been made in elucidating various aspects of this interaction, a comprehensive understanding of dynamic processes during fermentation including initial resistance to polysaccharide digestion, structural changes during hydrolysis, degradation mechanisms, resulting alterations in microbial communities and metabolite profiles, as well as their impacts on host health remains an area requiring requirement for systematic analysis and integration.

This review systematically examines the fermentation-driven interactions between plant polysaccharides and gut microbiota throughout the digestive process. We explore four key aspects: (1) Digestive characteristics of various plant polysaccharides in the upper digestive tract; (2) Microbial mechanisms driving polysaccharide fermentation; (3) Alterations in the physicochemical properties, structural composition, and bioactivity of polysaccharides caused by gut microbiota fermentation; (4) Effects of plant polysaccharide fermentation on gut microbiota composition, metabolites (particularly SCFAs and other key metabolites), and microbial functions. Additionally, we discuss the complex fermentation-driven dialogue’s impact on human health, particularly its regulatory roles in glucose-lipid metabolic homeostasis, immune function, cardiovascular system regulation, and gut-brain communication. This review aims to provide scientific evidence for understanding how plant polysaccharides promote gut health and overall wellness, while advancing their potential applications.

## 2 Methods

To identify relevant studies, we conducted comprehensive searches in Web of Science, PubMed, and Google Scholar using the keywords “plant polysaccharides, gut microbiota, fermentation, digestive properties, degradation mechanism, structural changes, microbiota regulation, health effects, SCFAs, CAZymes.” The search returned 3,212 records. Following screening, brief communications, retracted articles, and manuscripts unrelated to the interplay between plant polysaccharides, gut microbiota, and structural changes were excluded. Ultimately, high-quality publications from 2000 to 2025 addressing this topic were selected for discussion.

## 3 Digestive characteristics of plant polysaccharides in the gastrointestinal tract

The journey of dietary plant polysaccharides begins in the upper gastrointestinal tract (GI). By simulating their digestion processes through oral cavity, stomach, and small intestine, we can better understand the fate of these metabolites during initial transit through the body. This provides a basis for further investigation into the quantity and structural state of polysaccharides reaching the colon for microbial fermentation. In this context, “digestive properties” primarily refer to the resistance of these metabolites to host enzymatic degradation and harsh chemical environments in the digestive tract. These characteristics are typically assessed by monitoring key physicochemical properties of polysaccharides, such as molecular weight (MW) distribution, reducing sugar (CR) content, and solution conformation (Table 1). Generally, most non-starch plant polysaccharides demonstrate significant resistance during the upper gastrointestinal tract phase.

**TABLE 1:** Digestive characteristics of plant polysaccharides.

Name of polysaccharides	Source	Structure	*Mw*			C_R_			Conclusion	References
			Mouth	Stomach	Small intestine	Mouth	Stomach	Small intestine		
AnPs	*Ascophyllum nodosum*	-	NP	NP	NP	NP	NP	NP	Stabilization	Chen et al. (2018a)
CSPW	*Coix lacryma-jobi* L	-	NP	*	NP	NP	*	NP	Limited degradation	Xue et al. (2025)
JHP	*Coreopsis tinctoria* Nutt	-	NP	*	NP	NP	*	NP	Limited degradation	Wu et al. (2021c)
OPP	*Abelmoschus esculentus* (L.) Moench	-	NP	NP	NP	NP	*	NP	Limited degradation	Wu et al. (2021b)
LBPS	*Lycium barbarum*	-	NP	NP	NP	NP	NP	NP	Stabilization	Ding et al. (2019)
LLP	*Nelumbo nucifera* Gaertn	-	NP	NP	NP	NP	NP	NP	Stabilization	Wu et al. (2022b)
PSP	*Polygonatum spp*	-	NP	NP	NP	NP	NP	NP	Stabilization	Hu et al. (2024)
TSP	*Tamarindus indica* L	-	NP	NP	NP	NP	NP	NP	Stabilization	Li et al. (2020b)
LRP	*Nelumbo nucifera* Gaertn	Backbone of (1 → 4)-α-D-glucan with α-D-glycopyranosyl moieties connected to C-6 positions of the glucose residues as side chains approximately every six residues	NP	*	*	NP	*	*	Limited degradation	Guan et al. (2022)
JPS	*Ziziphus Jujuba cv. Pozao*	-	NP	*	NP	NP	*	NP	Limited degradation	Han et al. (2022)
TP	*Camellia sinensis* (L.) O. Kuntze	-	NP	NP	NP	NP	NP	NP	Stabilization	Tan et al. (2023)
CYP	*Dioscorea opposita* Thunb	-	NP	*	NP	NP	*	NP	Limited degradation	Bai et al. (2023)
CCPP	*Cyclocarya paliurus*	-	-	-	-	-	-	-	Stabilization	Yao et al. (2020)
LLP	*Eriobotrya japonica* (Thunb.) Lindl	-	NP	*	*	NP	*	*	Limited degradation	Wu et al. (2021a)
DGPN	*Panax ginseng* C. A. Meyer	Backbone consisting of →4)-α-Glcp-(1→, which was substituted at the O-6 positions	NP	NP	*	NP	NP	*	Limited degradation	Xu et al. (2025a)
ACS	*Areca catechu* L	Backbone of →4)α-D-Glcp-(1→, α-D-Glcp (1→, →3,4)α-D-Glcp-(1→ and →4)β-D-Glcp, and the side chain was consisted of α-D-Glcp-(1 →	NP	NP	NP	NP	NP	NP	Stabilization	Chen et al. (2010)
TFP	*Tremella fuciformis*	-	NP	NP	NP	NP	NP	NP	Stabilization	Wu et al. (2022a)
ST-P2	*Sargassum thunbergii*	The main linkage types were identified as (1 → 5)-α-L-Araf, (1 → 3)-α-L-Manp, (1 → 3,6)-β-D-Galp, (1 → 6)-α-D-Glcp, and (1 → 3)-β-D-Xylp	-	-	-	-	-	-	Stabilization	Fu et al. (2018)
PFP-T	*Passiflora edulis f. flavicarpa* L. peel	-	NP	NP	NP	NP	NP	NP	Stabilization	Yu et al. (2024)
LBPS	*Lycium barbarum* L	-	NP	NP	NP	NP	NP	NP	Stabilization	Gu et al. (2025)

No study; NP: No significant difference; *: Significant difference vs. previous stage (*p* < 0.05).

### 3.1 Limited degradation in the oral cavity

The oral cavity initiates mechanical decomposition through chewing and performs preliminary chemical digestion of polysaccharides via salivary amylase. However, salivary amylase primarily targets the hydrolysis of α-1,4 glycosidic bonds in starch. Most plant polysaccharides have complex structures. In addition to amylaceous polysaccharides, non-starch polysaccharides in plants exhibit diverse types of glycosidic bonds and complex branched structures, making them resistant to salivary amylase (Wu et al., 2021c). Studies on the digestibility of snow chrysanthemum polysaccharide (JHP), okra pectin polysaccharide (OPP), and barbary wolfberry polysaccharide (LBPS) also confirmed that these complex polysaccharides show no significant molecular weight changes after simulated salivary digestion and release minimal reducing sugars (Ding et al., 2019; Wu et al., 2021b; Wu et al., 2021c). Similarly, polysaccharides from lotus leaves, polygonatum, and rambutan remain stable during the salivary digestion phase (Li et al., 2020b; Wu et al., 2022b; Hu et al., 2024). This further confirms that most non-amyloid polysaccharides maintain stability during salivary digestion.

### 3.2 Partial hydrolysis in the stomach

After entering the stomach, polysaccharides not only encounter a highly acidic environment (pH 1.5–3.5) but also undergo enzymatic hydrolysis by pepsin and gastric lipase. However, these enzymes have limited ability to hydrolyze the glycosidic bonds of polysaccharides. However, low pH may induce the cleavage of acid-sensitive glycosidic bonds, particularly those in certain neutral polysaccharides that are easily hydrolyzed by gastric acid, leading to partial degradation of polysaccharides and a slight decrease in average molecular weight. For example, lotus root polysaccharide (LRP) showed a reduction in molecular weight from 15.74 kDa to 12.72 kDa during simulated gastric digestion, though no increase in reducing sugar content was observed. This reduction may be due to the cleavage of LRP glycosidic bonds caused by the gastric acid environment (Guan et al., 2022). Similarly, the polysaccharides JPS and JHP isolated from jujube and snow chrysanthemum also exhibited molecular weight reduction under gastric conditions (Han et al., 2022; Tan et al., 2023). However, this acid-catalyzed hydrolysis is generally limited, as the core structures of polysaccharides often remain largely intact (Wu et al., 2021c; Bai et al., 2023; Hu et al., 2024). Additionally, many structurally complex acidic polysaccharides such as Barbary wolfberry polysaccharide (LBPS), Chinese knotweed polysaccharide (CCPP), and papaya fruit pectin polysaccharide (PFP-T) demonstrated remarkable stability in gastric environments without significant changes (Ding et al., 2019; Yao et al., 2020; Zhang et al., 2022a).

### 3.3 Resistance and digestion in the small intestine

Small intestinal fluid contains various digestive enzymes, mainly including pancreatic enzymes (such as pancreatic amylase, trypsin, and lipase) and intestinal brush border membrane enzymes (such as maltase, sucrose, and lactase). These enzymes work synergistically to partially decompose polysaccharides in a neutral to weakly alkaline environment (pH7.0–8.0). Particularly, amyloid polysaccharides are often effectively hydrolyzed into monosaccharides here, but most non-starch plant polysaccharides continue to exhibit significant resistance due to the lack of appropriate host enzymes (Chen et al., 2018a; Ge et al., 2021; Wardman et al., 2022). Studies have found that during simulated intestinal digestion, specific polysaccharides such as de-starched ginseng polysaccharides (DGPNs), okra pectin (OPP), and those from chrysanthemum and loquat leaves undergo slight degradation, manifested as a further reduction in molecular weight or a minor increase in reducing sugars, which may be related to the disruption of glycosidic bonds by substances like pancreatic enzymes in intestinal fluid (Wu et al., 2021a; Wu et al., 2021b; Wu et al., 2021c; Xu et al., 2025a). However, many other polysaccharides, including lyceum chinensis (LBPS), fucoidan (AnPs), and polygonatum polysaccharide (PSP), demonstrate remarkable stability, with their molecular weight and reducing sugar content remaining essentially unchanged after simulated intestinal stages (Chen et al., 2018a; Ding et al., 2019; Hu et al., 2024).

### 3.4 Delivery to the colon for fermentation

In summary, most non-starch polysaccharides from plant sources undergo minimal modification during their passage through the oral cavity, stomach, and small intestine. While partial hydrolysis (primarily acid-driven in the stomach) may slightly reduce molecular weight, the basic structure of these polysaccharides remains largely intact. This inherent resistance to host digestion is crucial, as it ensures that most of these complex carbohydrates reach the colon intact. This provides essential substrates for the vast microbiota and lays the foundation for subsequent fermentation processes. Ultimately, this fermentation unleashes the prebiotic potential of polysaccharides, promoting overall health (Chen et al., 2010; Ge et al., 2024; Xue et al., 2025).

## 4 Fermentation characteristics of plant polysaccharides and microbiota interactions

**FIGURE 2:**
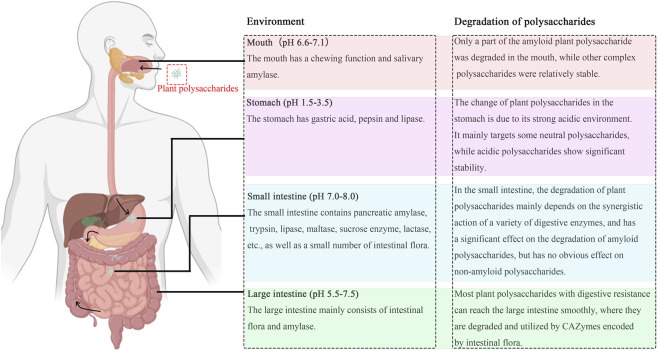
The journey of plant polysaccharides in the digestive tract.

After digestion in the upper gastrointestinal tract (as described in Section 2), digested plant polysaccharides reach the colon, marking the onset of a critical fermentation process. This biochemical process provides energy for complex gut microbiota while driving the conversion of these polysaccharides into absorbable metabolites, thereby influencing host health (Figure 2). Gut microbiota demonstrate remarkable adaptability and specificity in degrading and utilizing structurally diverse polysaccharides. This section will elucidate the fermentation characteristics of polysaccharides through three aspects: degradation mechanisms, structural modifications, and changes in gut microbiota and their metabolites, further exploring the interactions between polysaccharides and gut microbiota. Relevant literature has been compiled into Table 2.

**TABLE 2:** Fermentative characteristics of plant polysaccharides.

Name of polysaccharides	*Mw*	C_R_	Change of composition of monosaccharides	Change of functional group	Change of gut microbiota	Change of SCFAs	References
AnPs	*	*	-	-	Increased Bacteroidetes*,* Firmicutes	Increased Acetic acid, propionic acid	Chen et al. (2018a)
CSPW	*	*	-	-	Increased *Limosilicactobacillus, Bifidobacterium, Collinsella*	Increased Acetic acid, Propionic acid, n-Butyric acid	Xue et al. (2025)
JHP	*	*	Increased Glucose, Glucuronic acid, Rhamnose, Mannose Decreased Galacturonic acid, Galactose, Xylose	Vanished 1743 cm-1 C=O contraction vibration peak	Increased *Bifidobacterium, Lactobacillus, Megamonas, Megasphaera* Decreased *Fusobacterium, Klebsiella,Escherichia-Shigella*	Increased Acetic acid, Propionic acid, n-Butyric acid, n-Valeric acid	Wu et al. (2021c)
OPP	*	*	Increased Galactose Decreased Galacturonic acid	NP	Increased *Phascolarctobacterium, Megasphaera, Lachnoclostridium, Desulfovibrio* Decreased *Bilophila, Fusobacterium*	Increased Acetic acid, Propionic acid	Wu et al. (2021b)
LBPS	*	*	Decreased Glucose, Glucuronic acid, Galacturonic acid	-	Increased *Bifidobacterium, Bacteroides*	Increased Acetic acid, Propionic acid, n-Butyric acid	Ding et al. (2019)
LLP	*	*	Decreased Arabinose, Galactose, Galacturonic acid	NP	Increased *Bacteroides, Bifidobacterium, Megamonas, Collinsella*	Increased Acetic acid, Propionic acid, i-Butyric acid	Wu et al. (2022b)
PSP	*	*	Increased Mannose, Rhamnose Decreased Glucuronic acid, Galacturonic acid	NP	Increased *Bacteroides, Clostridium sensu stricto* Decreased Firmicutes	Increased Acetic acid, Propionic acid	Hu et al. (2024)
TSP	*	*	-	-	Increased *Lactobacillus, Parabacteroides, Prevotella, Faecalibacterium* Decreased *Escherichia-Shigell, Dorea*	Increased Acetic acid, Propionic acid,n-Butyric acid	Li et al. (2020b)
LRP	*	*	-	-	Increased Bacteroidetes*, Escherichia–Shigella, Megamonas* Decreased Firmicutes	Increased Acetic acid, Propionic acid,n-Butyric acid	Guan et al. (2022)
JPS	*	*	Increased Glucuronic acid Decreased Mannose, Rhamnose, Arabinose, Galactose, Glucose	-	Increased *Megasphaera, unclassified_f_Veillonellaceae* Decreased *Bacteroides, Lachnoclostridium, Parabacteroides, Sutterella, Lachnospiraceae UCG-010, Butyicimonas*	Increased Acetic acid, Propionic acid,n-Butyric acid	Han et al. (2022)
TP	*	*	Increased Rhamnose Decreased Galactose, Arabinose	Increased 3420 cm-1 hydroxyl O-H stretching vibration peak	Increased *Bifidobacterium, Prevotella Phascolarctobacterium*	Increased Acetic acid, Propionic acid, n-Butyric acid	Tan et al. (2023)
CYP	*	*	-	-	Increased *Enterococcus, Bifidobacterium, Bifidobacterium* *Lactobacillus, Dialister, Olsenella, Mitsuokella*	Increased Acetic acid, Propionic acid,n-Butyric acid	Bai et al. (2023)
CCPP	-	-	-	-	Increased *Ruminococcus_bromii, Anaerotruncus_colihominis, Clostridium_methylpentosum, Roseburia_intestinalis, Roseburia_hominis, Clostridium_asparagiforme, Pseudoflavonifractor_capillosus, Intestinimonas_butyriciproducens, Intestinimonas_*sp.*_GD2, Oscillibacter_valericigenes, Oscillibacter_ruminantium*	Increased Acetic acid, Propionic acid, n-Butyric acid, n-Valeric acid	Yao et al. (2020)
LLP	*	*	Increased Glucuronic acid Decreased Galactose, Arabinose, Galacturonic acid	NP	Increased *Megasphaera, Megamonas, Bifidobacterium, Phascolarctobacterium, Desulfovibrio*	Increased Acetic acid, Propionic acid, n-Butyric acid	Wu et al. (2021a)
DGPN	*	*	Increased Galactose, Arabinose Decreased Glucose	NP	Increased *Bacteroides, [Eubacterium]_nodatum_group, Ligilactobacillus, Enterococcus* Decreased *Escherichia_Shigela*	Increased Acetic acid, Propionic acid	Xu et al. (2025a)
ACS	-	-	-	-	Increased Bacteroidota, Gram-positive bacteria Decreased Firmicutes*,* Proteobacteria	Increased Acetic acid, Propionic acid	Chen et al. (2010)
TFP	*	*	Decreased Fucose, Xylose	Decreased 1729 cm-1 C=O contraction vibration peak	Increased *Phascolarctobacterium, Bacteroides, Lachnoclostridium*	Increased Acetic acid, Propionic acid, n-Butyric acid, n-Valeric acid	Wu et al. (2022a)
Blackberry polysaccharides BBP	-	-	-	-	Increased *Oscillospira, Bacteroidaceae, Bacteroides* Decreased *Allobaculum*	Increased Acetic acid, Propionic acid,n-Butyric acid	Xi et al. (2024)
*Polygonatum sibiricum Red* leaves Polysaccharides PsPs	-	-	-	-	Increased Firmicutes*, Lactobacillus* Decreased Bacteroidetes*, Lachnospiraceae, Bacteroides, Muribaculaceae, Alistipes, Odoribacter*	Increased Acetic acid, Propionic acid,n-Butyric acid, i-Butyric acid, i-Valeric acid	Luo et al. (2022)
ST-P2	*	*	Decreased Galacturonic acid, Mannose, Arabinose, Galactose	-	Increased Bacteroidetes*, Faecalibacterium, Coprococcus, Bifidobacterium, Coprococcus, Parasutterella* Decreased Firmicutes	Increased Acetic acid, Propionic acid, n-Butyric acid, n-Valeric acid	Fu et al. (2018)
PFP-T	*	*	Increased Galacturonic acid Decreased Mannose, Rhamnose, Arabinose, Galactose, Glucose	Vanished 1015 and 967 cm^−1^ absorption peaks of the RG-I	Increased *Klebsiella, Megasphaera, Dialister, f__Veillonellaceae_Unclassified, Prevotella,* Negativicute*,* Firmicutes*, o__Veillonellales_Selenomonadales,* k__Bacteria*, Megasphaera, Dialister*	Increased Acetic acid, Propionic acid,n-Butyric acid	Yu et al. (2024)
LBPS	*	*	Decreased Glucose, Mannose	-	Increased *Bacteroides, Faecalibacterium, Blautia, Fusicatenibacter*	Increased Acetic acid, Propionic acid,n-Butyric acid	Gu et al., (2025)

No study; NP: no significant difference; *: Significant difference vs. initial stage (*p* < 0.05).

### 4.1 Key microbial mechanisms driving Polysaccharide fermentation

The efficient degradation and utilization of plant polysaccharides depends on complex microbial strategies, mainly involving specific enzyme systems and synergistic metabolic interactions.

#### 4.1.1 Enzyme systems: CAZymes and PULs

The core enzyme system consists of carbohydrate active enzymes (CAZymes), and the key families involved in polysaccharide decomposition include:

Glycoside hydrolases (GHs): According to the latest update of the Carbohydrate Active Enzymes (CAZy) database, there are 141 known GH families. Over 80% of these GH families directly or indirectly participate in polysaccharide degradation catalysis by hydrolyzing glycosidic bonds. For homogeneous polysaccharides, such as cellulose containing β-1,4-glycosidic bonds in D-glucose units, the core enzymes for degradation include GH1 (β-glucosidase), GH5, GH6, and GH7 families (e.g., endoglucanase, cellobiohydrolase) (Lombard et al., 2014; Crost et al., 2023). The degradation of more complex β-glucans primarily relies on GH9 (endogluco-β-1,4-glucosidase) and GH16 families (Drula et al., 2022). Starch contains not only amylose linked by α-1,4-glycosidic bonds but also amylopectin connected through α-1,6-glycosidic bonds, both of which depend on GH13 families (α-amylase, amylopectinase) for degradation (Holscher, 2017). Hemicellulose, a complex polysaccharide composed of various monosaccharides like xylan and arabinoglucan, is mainly degraded by GH10/11 families (xylanase) and GH43/51/54 families (arabinofuranoside enzymes) (Flint et al., 2012). Pectin, as a highly complex class of plant polysaccharides, demonstrates distinct degradation mechanisms depending on its homologous galacturonan (HG) type. Homologous galacturonan (HG) pectin is primarily degraded by GH28 family enzymes (endogalactanase and exogalactanase) through cleavage of α-1,4-glycosidic bonds. Rhamnogalacturonan-I (RG-I) pectin requires GH28 family enzymes (endogalactanase, exogalactanase, and rhamnogalacturonase) to break both α-1,4-glycosidic and α-1,2-glycosidic bonds. In contrast, Rhamnogalacturonan-II (RG-II) pectin depends on multiple GH enzymes including GH43/51 (arabinofuranosidease), GH53 (galactanase), GH78 (β-galactosidase), and GH95 family (α-galactosidase) (Gao et al., 2024). GHs play a crucial role in polysaccharide degradation with distinct specificity: homogeneous polysaccharides can typically be effectively degraded by a single enzyme, while complex polysaccharides containing multiple glycosidic bonds require coordinated action of various GHs to achieve complete breakdown.

Polysaccharide Lyases (PLs): The CAZy database currently contains 45 PL families, all of which directly participate in polysaccharide degradation through β-elimination mechanisms that cleave glycosidic bonds, primarily targeting polygalacturonic acid-containing polysaccharides. Families PL1 (pectin acid lyase), PL2 (polymethylgalacturonic acid lyase), PL9, and PL11 (polymannuronic acid enzymes) are mainly responsible for pectin degradation. Families PL6 (fucoidan lyase), PL7, and PL13 primarily act on algal polysaccharides such as fucoidan (Despres et al., 2016; Drula et al., 2022). The PL15 family targets the α-1,4 glycosidic bond in pectin and performs deacetylation (Drula et al., 2022). Cellulose degradation depends on the PL9 family (cellulose lyases), while fucoidan degradation relies on the PL17 family (fucoidan lyases).

Carbohydrate Esterases (CEs): There are 21 known families of CEs that directly participate in polysaccharide degradation. Their primary function is to remove ester modifications such as acetyl, methyl, or ferulate groups (e.g., CE1 acting on xylan (Flint et al., 2012)) or sulfate groups from polysaccharide chains, thereby enhancing the accessibility of glycosidic bonds (GHs/PLs) and altering polysaccharide properties (Moraïs et al., 2024; Martens et al., 2009; McKee et al., 2021). For instance, during xylan degradation, CE1 (Acetylxylan esterase) and CE16 family members (α-glucuronidase) cleave acetyl groups on the main chain and 4-O-methylglucuronate bonds on side chains, exposing more glycosidic bonds to facilitate complete polysaccharide breakdown. For pectin containing methyl ester groups, CE4 family members (Pectin methylesterase) remove these methyl ester groups, making it easier for GHs and PLs to further degrade the material.

Glycosidase (GTs): The function of GT is completely opposite to that of GH. They are mainly involved in the synthesis of glycosidic bonds between two monomeric units.

Auxiliary active enzymes (AAs): participate in lignocellulose degradation and often work in synergy with CAZymes.

**FIGURE 3:**
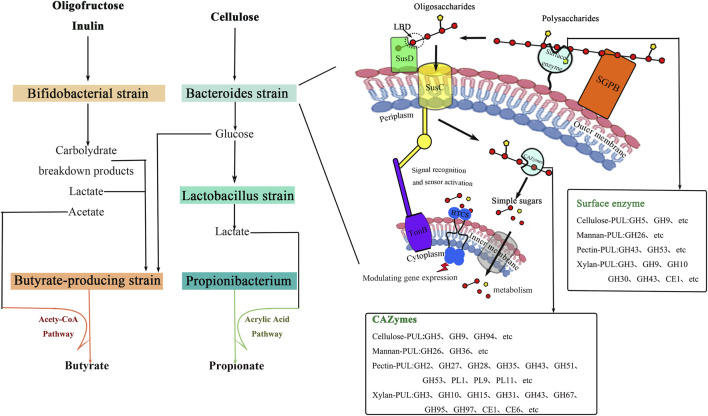
Cross-feeding effect of intestinal microbiota and the core mechanism of polysaccharide fermentation by *Bacteroides*.

PULs are gene clusters in microbial genomes such as Bacteroidetes that encode enzymes and transport proteins for polysaccharide degradation, responsible for the recognition, degradation, and metabolism of plant polysaccharides (Kaoutari et al., 2013; Beidler et al., 2023). In human gut microbiota, Bacteroidetes and Firmicutes dominate. *Bacteroides* species utilize PULs to encode CAZymes enzyme systems that specifically cleave polysaccharide chains, enabling modular specialization in polysaccharide degradation. The structural and functional diversity of PULs allows them to adapt to various polysaccharide substrates across ecological niches, serving as the core driver of microbial carbon metabolism. As shown in Figure 3, complex plant polysaccharides are degraded into oligosaccharide fragments through binding to surface glycoprotein-binding domains (SGPBs) on *Bacteroides* cell membranes and enzymatic cleavage. These oligosaccharides are recognized by the ligand-binding domain (LBD) of TonB-dependent transporters (SusC/D), enabling their entry into the periplasmic space. Within this environment, they are further processed by enzymes such as GHs, PLs, and CEs, ultimately yielding simple sugars (Despres et al., 2016; McKee et al., 2021). These simple sugars are transported into the cytoplasm for metabolism through specialized co-transporters on the inner membrane. The expression of PULs is dynamically regulated by RNA-binding proteins and heterogeneous non-coding RNAs. Degradation of these sugars activates HTCS-like regulators, triggering a cascade of signaling events that induce the expression of polysaccharide utilization genes to adapt to fluctuations in the body’s nutrient levels (Adams Amanda et al., 2021). To date, studies on Bacteroidetes ‘degradation of various pectin types have yielded significant insights. In pectin degradation, *Bacteroides* employs PUL49 and PUL50 to target homogalacturonic acid (HG) and type I rhamnoglucuronidase (RGI), respectively. PUL49 encodes Polysaccharide Lyases (PL1, PL9) and GHs (GH28), responsible for cleaving HG’s main chain. PUL50 contains GH88 and CE12 family enzymes that perform de-esterification and hydrolysis of RGI side chains (Despres et al., 2016). This modular design enables PULs to efficiently degrade plant polysaccharides with strong substrate specificity. After degradation, plant polysaccharides are converted into monosaccharides, which further ferment into metabolites like SCFAs. For instance, AnPs, a seaweed-derived polysaccharide, significantly increases *Bacteroides* abundance in in vitro colon models while promoting short-chain fatty acid production, particularly acetic and propionic acids (Chen et al., 2018a). In summary, PULs serve as a “molecular toolkit” for gut microbiota to degrade plant polysaccharides. Through substrate-specific degradation, metabolite generation, and microbial regulation, they play a central role in ecological adaptation and host health. In summary, different families of carbohydrate-active enzymes (CAZymes) perform distinct functions and act synergistically. As a “molecular toolbox”, PULs enable the specific degradation and metabolism of plant polysaccharides. Together, they constitute the core mechanism underlying polysaccharide utilization by the gut microbiota.However, current research on variations in carbohydrate-active enzyme profiles among individuals, host genetics, and other factors remains insufficient. There is also a lack of attention to the heterogeneity in CAZyme expression, PUL abundance, and regulatory patterns across different individuals. This limits the development of personalized nutritional strategies and precision prebiotics.

#### 4.1.2 Cross-feeding effects

The degradation of plant polysaccharides in the colon not only involves direct utilization by specific bacterial communities but may also require intercolonic collaboration. Research has confirmed this phenomenon through cross-feeding effects among gut microbiota. Cross-feeding (Figure 3) refers to a metabolic complementarity mechanism where different microorganisms achieve nutritional synergy and co-growth through shared metabolites, playing a crucial role in maintaining gut microbiota stability and functionality (Heintz-Buschart and Wilmes, 2018; Payling et al., 2020). This mechanism can be categorized into three types based on metabolite flow direction, microbial reciprocity levels, and metabolite properties: substrate cascade metabolism, metabolite exchange, and vitamin sharing.

Substrate-coupled catabolism is a highly efficient metabolic process that facilitates orderly substrate-to-product conversion through coordinated enzymatic systems and efficient intermediate transport among microbial communities, playing a crucial role in cellular energy metabolism. During plant polysaccharide fermentation, primary-degrading bacteria (e.g., *Bacteroides*) break down polysaccharides into oligosaccharide fragments for secondary bacteria (e.g., *Bifidobacterium*, Firmicutes) to further metabolize (Fernandez-Julia et al., 2021). For example, *Bacteroides* degrades cellulose to produce glucose, while butyrate-producing bacteria (e.g., Rosetta spp.) utilize glucose to generate butyrate (Belenguer et al., 2006). *Bacteroides* cellulosilyticus degrades arabinoglucan (AGs) in plant cell walls by secreting GHs (e.g., GH30 and GH157 family enzymes) to cleave polysaccharide side chains, releasing oligosaccharides such as β-1,3-galactose-2,6-diphosphate/3-phosphogluco-1,3-lactose (Munoz et al., 2020). These oligosaccharides remain incompletely utilized by primary-degrading *Bacteroides* but serve as substrates for secondary fermenters like *Bifidobacterium* breve UCC2003. Through its bga gene cluster (encoding oligosaccharide transporters and GHs), *Bifidobacterium* breve UCC2003 metabolizes these oligosaccharides, enabling cross-species carbon resource sharing (Fernandez-Julia et al., 2023).

Metabolites exchange refers to the process of utilizing metabolites between microbial communities. For example, dietary fiber fermented by *Bifidobacterium* produces acetic acid, which is then utilized by butyrate-producing bacteria that generate butyrate through the acetyl-CoA pathway, thereby enabling the exchange of metabolic products between different bacterial groups. Additionally, *Bifidobacterium* adolescentis (adolescent bifidobacteria) can degrade plant starch or fructooligosaccharides (FOS), producing lactic acid and acetic acid as primary metabolites. These products are further utilized by butyrate-producing bacteria such as Eubacterium hallii or Anaerostipes caccae, which convert them into butyrate via the acetyl-CoA pathway (Belenguer et al., 2006).

Vitamin sharing in cross-feeding primarily manifests through bacterial exchange of metabolites within microbial communities, addressing nutritional deficiencies and enhancing community stability. Studies indicate that the metabolites of vitamin B complexes (e.g., B1, B6, B12) are shared among gut microbes, which helps maintain both microbial stability and host health. Notably, *Bacteroides* species synthesize vitamin B12 that can be utilized by other bacterial groups (Magnúsdóttir et al., 2015).

In nature, the degradation of most plant polysaccharides likely relies primarily on these collaborative mechanisms. The existence of such complex degradation processes enhances substrate utilization efficiency while strengthening intermicrobial dependencies. This not only promotes the stability and diversity of gut microbiota but also broadens the range of fermentation end products. Ultimately, this significantly boosts the degradation and utilization of substrates like polysaccharides, maintaining gut health and supporting overall wellbeing. More importantly, it provides a theoretical foundation for developing prebiotics.

### 4.2 Degradation and utilization of plant polysaccharides by microorganisms in fermentation

The fermentation of plant polysaccharides by gut microbiota triggers a cascade of physicochemical and structural changes mediated by microbe-specific enzymes. These structural modifications are critical for the efficient utilization of polysaccharides by microbes and determine their potential downstream bioactivity.

#### 4.2.1 Changes in viscosity and solubility

The viscosity of polysaccharides is primarily determined by their molecular weight, chain conformation, and branching degree. Studies indicate that polysaccharide viscosity significantly decreases during fermentation, mainly due to CAZymes disrupting glycosidic bonds. GHs and Polysaccharide Lyases (PLs) can cleave glycosidic bonds in both main and side chains of polysaccharides, thereby reducing their polymerization degree (Kaoutari et al., 2013; Wu et al., 2021b). For instance, *Bacteroides* species utilize enzyme systems encoded by PULs to efficiently degrade large molecular polymers like pectin into smaller fragments, significantly lowering solution viscosity (Martens et al., 2009; Palevich et al., 2019; Wu et al., 2021b). This viscosity reduction affects intestinal transit time and nutrient absorption dynamics.

The solubility of polysaccharides typically increases during fermentation. Firstly, the complex structures of polysaccharides are disrupted during hydrolysis, thereby reducing intermolecular forces and steric hindrance. Additionally, microbial-encoded CEs can remove hydrophobic modifying groups such as methyl or acetyl groups from polysaccharides (e.g., those in xylan or xylocellulose), enhancing their hydrophilicity (Moraïs et al., 2024; Suez et al., 2022). Notably, enzymatic cleavage of polysaccharides generates more free hydroxyl groups, further boosting solubility. Furthermore, SCFAs produced during fermentation may promote the dissolution of certain polysaccharides by lowering intestinal pH.

#### 4.2.2 Dynamic changes in total sugar and reducing sugar

The change of total sugar and reducing sugar concentration in the fermentation process is an important index to evaluate the fermentation process. The change of reducing sugar content reflects the degradation and utilization efficiency of the microbial flora to polysaccharides. In the initial stage, enzymatic hydrolysis by microbial glycosidases (GHs, PLs) cleaves glycosidic bonds, releasing oligosaccharides and monosaccharides that temporarily elevate reducing sugar content (Wu et al., 2021b; Wu et al., 2022a), indicating polysaccharide degradation. However, as fermentation progresses, reducing sugar levels in the culture broth gradually decrease. This occurs because microorganisms increasingly utilize released sugars as their primary carbon and energy sources for growth and metabolism, with consumption rates exceeding production rates (Wu et al., 2021b; Ke et al., 2023; Hu et al., 2024). Additionally, the magnitude and rate of total sugar reduction serve as another key indicator of microbial utilization efficiency. For instance, during the hydrolysis of okra pectin (OPP), tremella polysaccharide (TFP), and polygonatum polysaccharide (PSP), significant reductions in total sugar content demonstrate that gut microbiota exhibit exceptional efficiency in degrading and utilizing indigestible polysaccharides, ultimately producing SCFAs (Wu et al., 2021b; Wu et al., 2022a; Hu et al., 2024). Compared to complex insoluble polysaccharides, gut microbiota typically degrade and utilize soluble simple polysaccharides like inulin more rapidly.

#### 4.2.3 Changes in molecular weight and monosaccharide composition

A defining feature of polysaccharide fermentation is the significant reduction in MW. Microbiota-encoded glycosidases (GHs) and proteases (PLs) systematically cleave sugar chains to produce smaller oligosaccharides that ultimately form monosaccharides (Wu et al., 2022b). This molecular weight reduction is crucial for maintaining the biological activity of polysaccharides. Lower molecular weight fragments (e.g., <10 kDa) exhibit reduced steric hindrance, potentially enhancing bioavailability or exhibiting unique biological properties (Hu et al., 2018; Xi et al., 2024). Studies demonstrate that low-molecular-weight plantain polysaccharides effectively promote *Lactobacillus* proliferation and short-chain fatty acid (SCFAs) production (Hu et al., 2018).

The monosaccharide composition serves as a key indicator for characterizing polysaccharide structures, where the relative proportions of individual sugars reflect the inherent architecture. Changes in monosaccharides during fermentation demonstrate microbial preferences for specific degradation pathways and the specificity of encoded enzymes. For instance, *Bacteroides* species predominantly degrade complex polysaccharides like pectin and hemicellulose (Comtet-Marre et al., 2017), while Firmicutes are more involved in breaking down starch or cellulose (Crost et al., 2023; Gao et al., 2024). Specific enzymes determine which bonds are cleaved and which sugars are preferentially released: galacturonic acid hydrolases (GH28) and arabinofuranosidase (GH43) target pectin to release galacturonic acid and arabinose (Flint et al., 2012); while xylanases (GH10/11) and related CE/Arabinofuranosidase enzymes (GH51/54) extract xylose and arabinose from arabinoglucan (Lin et al., 2023). Notably, during fermentation of lotus leaf polysaccharides, significant changes occur in the ratio of arabinose, galactose, and galacturonic acid (Wu et al., 2022b), highlighting the selective enzymatic activity of microbial systems.

### 4.3 Changes in biological activities of plant polysaccharides during digestion and fermentation

The digestive and hydrolysis processes of plant polysaccharides can alter their biological activities such as antioxidant, antitumor, immunomodulatory, antibacterial, and anti-inflammatory effects (Wang et al., 2024a). This is mainly due to the changes in structural characteristics such as molecular weight, monosaccharide composition, functional groups, and conformation of the polysaccharides caused by enzymatic hydrolysis.

#### 4.3.1 Effects of molecular weight on biological activity

The reduction of molecular weight is the most important structural change of plant polysaccharides during fermentation. This may serve as an important driver in the enhancement of most biological activities.

First, changes in molecular weight may improve the bioavailability of polysaccharides and potentially increase their direct contact with intracellular targets. Due to their large molecular size, high-molecular-weight polysaccharides can hardly pass through the intestinal mucosal barrier (including the physical barrier formed by intestinal epithelial cells and the biological barrier constituted by gut microbiota), and most can only exert local effects in the colon. In contrast, low-molecular-weight polysaccharide fragments (MW < 10 kDa) can more readily cross the tight junctions of intestinal epithelial cells or be absorbed via endocytosis, enter the systemic circulation, and reach various tissues and organs, thereby exerting systemic biological effects (Hu et al., 2018; Xi et al., 2024). For example, fermented low-molecular-weight acidic heteropolysaccharides from *Astragalus membranaceus* can enter the liver through the portal vein, activate the Nrf2/ARE signaling pathway in hepatocytes, and upregulate the expression of antioxidant enzymes such as superoxide dismutase (SOD), catalase (CAT), and glutathione peroxidase (GSH-Px), thereby exerting antioxidant effects and alleviating hepatic oxidative stress (Zhang et al., 2024a). Low-molecular-weight fermented *Glycyrrhiza* leaf polysaccharides (GLP) contain pyranose rings and α-glycosidic bonds. They can enter the blood circulation and reach zebrafish embryos, activate the Keap1/Nrf2 signalling pathway, and protect against AAPH-induced oxidative stress (Du et al., 2025).

Second, alterations in molecular weight may improve receptor binding affinity and promote the activation of downstream signalling pathways. The biological activities of plant polysaccharides (especially immunomodulatory activity) are mainly exerted by binding to pattern recognition receptors (PRRs) on the surface of immune cells (e.g., macrophages, dendritic cells), such as Toll-like receptors (TLRs), C-type lectin receptors (CLRs), and NOD-like receptors (NLRs). High molecular weight polysaccharides have a rigid chain conformation and a small number of exposed receptor binding sites due to steric hindrance, resulting in low receptor binding affinity; while the molecular weight reduction makes the polysaccharide chain conformation more flexible, exposes more binding sites (e.g., hydroxyl, carboxyl, sulfate groups), and significantly increases the binding affinity with PRRs (Xu et al., 2025a; Li et al., 2024a). For example, de-starched ginseng polysaccharides (DGPNs) after fermentation have a reduced molecular weight and an increased AG domain exposure, which can more effectively bind to TLR4 on the surface of macrophages, activate the TLR4/Myd88/NF-κB signal pathway, and promote the secretion of inflammatory cytokines (IL-6, TNF-α) and nitric oxide (NO), thus enhancing the immunomodulatory activity (Xu et al., 2025a); fermented lentinan has a reduced molecular weight and can bind to Dectin-1 (a CLR family receptor) on the surface of RAW264.7 macrophages with higher affinity, activate the Syk/NF-κB signal pathway, and inhibit the production of LPS-induced pro-inflammatory factors (Li et al., 2024a). However, most studies confirm the receptor binding and signal pathway activation of polysaccharides *in vitro* cell models, and lack the verification of the actual binding targets and signal pathway regulation of polysaccharides in animal and human bodies, which leads to the poor translatability of the research results.

Third, alterations in molecular weight may increase free radical scavenging ability via the availability of more active sites. The antioxidant activity of plant polysaccharides is mainly achieved by scavenging free radicals (DPPH, ABTS, hydroxyl, superoxide anion) through their active groups (e.g., hydroxyl, carboxyl)(Sun et al., 2020). For example, the molecular weight of Polygonatum polysaccharide (PSP) decreases from 87.6 kDa to 9.2 kDa after 24 h of fermentation, and the number of exposed hydroxyl groups increases by 2.3 times, leading to a significant increase in the scavenging rate of DPPH and superoxide radicals (Qi et al., 2024); the molecular weight of *Nostoc commune* Vauch. polysaccharide (NCVP) is reduced by half after fermentation, and the exposed carboxyl groups increase by 1.8 times, enhancing the ABTS and hydroxyl radical scavenging capacity (Li et al., 2022c). Although Qi and Li’s research confirmed that a reduction in molecular weight affects the antioxidant activity of plant polysaccharides, the specific changes in active groups such as hydroxyl and carboxyl groups responsible for their activity remain to be supported by further experimental data.High molecular weight polysaccharides have a compact molecular structure, and most of the active groups are wrapped in the molecule and cannot contact with free radicals; while the molecular weight reduction leads to the cleavage of the molecular chain, exposing a large number of active groups on the surface of the molecule, and the specific surface area is significantly increased, thus greatly enhancing the free radical scavenging ability (Yuan et al., 2020; Qi et al., 2024).

#### 4.3.2 Effects of monosaccharide composition on biological activity

The monosaccharide composition of polysaccharides is the basis of their structural characteristics, and its alteration during fermentation is another important factor affecting the biological activity of polysaccharides. The causal mechanism between monosaccharide composition alteration and biological activity change is mainly reflected in two aspects: the change of active centre structure and the change of receptor binding specificity, which is closely related to the substrate specificity of microbial enzyme systems.

First, change of active center structure and its impact on biological activity. Most plant polysaccharides have specific active structural domains (active centers), which are composed of specific monosaccharides connected by specific glycosidic bonds, the alteration of monosaccharide composition during fermentation directly changes the structure of the active centre, thus affecting the biological activity of polysaccharides. A representative example is the arabinogalactan (AG) domain, which is considered a core active centre of many plant polysaccharides (e.g., ginseng polysaccharide, longan polysaccharide), and its content and structure are thought to play a key role in modulating the immunomodulatory activity of polysaccharides (Hu et al., 2022; Xu et al., 2025a). Xu et al. investigated de-starched ginseng polysaccharides (DGPNs) mainly composed of glucose (96%) and found that fermentation led to a 36.54% decrease in glucose content, along with relative increases in arabinose and galactose proportions, which contributed to a higher proportion of AG domains. Subsequent studies demonstrated that fermented DGPNs exhibited more potent activation of the TLR4/Myd88 signaling pathway in macrophages, accompanied by enhanced immunomodulatory activity (Xu et al., 2025a). It is therefore speculated that microbial enzymes produced during fermentation may selectively degrade polysaccharide side chains (e.g., glucosyl side chains), thereby facilitating the relative enrichment of AG domains. Such structural alterations might serve as a key contributor to the improved immunomodulatory activity of fermented DGPNs. Hu et al. also observed in their study on longan polysaccharide (LP) that fermentation led to a decrease in glucose content, accompanied by relatively increased levels of arabinose and rhamnose, resulting in an elevated relative proportion of the arabinogalactan (AG) domain within the polysaccharide. Moreover, comparison between polysaccharide samples before and after fermentation revealed that fermented LP exhibited stronger activation effects on macrophages, with enhanced secretion levels of NO, IL-6 and TNF-α, indicating favourable immunomodulatory activity. Accordingly, *in vitro* simulated fermentation may promote an increased proportion of the AG domain via side-chain cleavage, thereby influencing the inherent immunomodulatory activity of the polysaccharide (Hu et al., 2022). Rhamnogalacturonan-I (RG-I) represents a characteristic structural domain of pectic polysaccharides; it is an acidic heteropolysaccharide composed of alternating rhamnose and galacturonic acid residues, and this domain has been reported to be closely associated with the bioactivity of pectic polysaccharides. Okra pectin (OPP) possesses a typical RG-I domain. After fermentation, the contents of rhamnose and galactose are increased, leading to an elevated content of the RG-I domain and optimized intestinal protective effects (Wu et al., 2021b). Existing research suggests that changes in structural domains may be potentially associated with enhanced polysaccharide activity. However, whether a quantitative relationship exists between changes in structural domains and the magnitude of biological activity remains unclear. Furthermore, the exact active sites where structural domains exert their biological effects, as well as their downstream signalling pathways, still need to be further verified.

Second, change of receptor binding specificity and its regulation of biological activity type. Different monosaccharide compositions determine the different receptor binding specificities of polysaccharides, the alteration of monosaccharide composition during fermentation can change the type of PRRs on the surface of immune cells bound by polysaccharides, thus regulating the type and intensity of biological activity. For example,: some polysaccharides with high glucose content can activate immune cells such as macrophages and dendritic cells through receptors including TLR4 and Dectin-1, thereby participating in immune regulation (Zhao et al., 2025; Gao et al., 2024); Some polysaccharides rich in galactose and arabinose may recognize receptors on monocytes and macrophages via pattern recognition receptors such as TLR2 and mannose receptor (MR), thereby contributing to the regulation of inflammatory homeostasis in the body (Newman et al., 2025; Yang et al., 2025a); Some polysaccharides with high uronic acid content may bind to members of the C-type lectin receptor (CLRs) family on immune cells such as macrophages and dendritic cells, and often exhibit antioxidant-dominant biological activities (Minzanova et al., 2018). For example, fermented lentinan has a decrease in glucose content from 76.9% to 50.5% and an increase in galactose content, leading to a change in its receptor binding specificity from TLR4 to TLR2; the binding to TLR2 can inhibit the LPS-induced NF-κB signal pathway, reduce the production of NO and IL-1β, and thus exert an anti-inflammatory effect (Li et al., 2024a). However, most current studies only reveal the general relationship between monosaccharide composition and receptor binding, while the underlying molecular mechanisms by which fermentation specifically affects receptor recognition remain unclear. In addition, most relevant conclusions are drawn from cell experiments, and sufficient verification is still lacking regarding their actual effects *in vivo* and stability across individual differences.

#### 4.3.3 Effects of conformation and morphology on biological activity

In addition to molecular weight reduction and monosaccharide composition change, the fermentation process also causes the conformation alteration of plant polysaccharides (including chain conformation and solid-state morphology), which indirectly regulates the biological activity of polysaccharides by changing the surface physicochemical properties (e.g., surface charge, hydrophilicity, specific surface area) of polysaccharides.

Chain conformation change: from rigid chain to flexible random coil. High molecular weight plant polysaccharides typically have a rigid chain conformation (e.g., triple helix, extended chain) due to intermolecular hydrogen bonds and steric hindrance; during fermentation, the cleavage of glycosidic bonds breaks the intermolecular hydrogen bonds, and the chain conformation changes from a rigid chain to a flexible random coil (Han et al., 2022; Yang et al., 2023). The flexible random coil conformation has the following advantages for the exertion of biological activity: (1) It increases the number of exposed active groups and receptor binding sites; (2) It improves the solubility and bioavailability of polysaccharides; (3) It makes the polysaccharide molecule more easily to enter the cell through endocytosis and exert intracellular biological effects. For example, red date polysaccharide (JPS) changes from a rigid extended chain to a flexible random coil after fermentation, and its solubility and receptor binding sites are significantly increased, thus enhancing its immunomodulatory activity (Han et al., 2022); *Polygonatum kingianum* polysaccharide (PKPS) changes from a rigid triple helix to a flexible random coil after fermentation, and its ability to bind to macrophages is significantly enhanced, thus improving its anti-aging activity (Yang et al., 2023).

Solid-state morphology change: from compact block/flake to loose irregular fragments. The solid-state morphology of plant polysaccharides also changes significantly during fermentation,the original compact block or flake morphology is transformed into loose irregular fragments due to the cleavage of the molecular chain (Han et al., 2022; Yang et al., 2023). This morphological change leads to a significant increase in the specific surface area of polysaccharides, which makes them more easily to contact with free radicals, immune cells and other targets, thus enhancing the biological activity. For example, red date polysaccharide (JPS) changes from a smooth flake-like morphology to irregular fragments after digestion and fermentation, leading to an increase in its immunomodulatory activity (Han et al., 2022); *Polygonatum kingianum* polysaccharide (PKPS) changes from a porous block-like structure to smooth irregular fragments after fermentation, thus enhancing its anti-aging activity (Yang et al., 2023).

However, most current studies on plant polysaccharides remain at the correlational level, merely reporting simple associations between structural modifications and enhanced bioactivities, without in-depth exploration of the underlying causal mechanisms. This study is similarly limited: it mainly focuses on the effect of a single structural change, i.e., molecular weight reduction, on biological activity, and fails to fully elucidate the synergistic effects of multiple structural modifications, including molecular weight reduction, monosaccharide composition changes, and higher-order conformational alterations. Consequently, the structure–activity relationship analysis is not sufficiently systematic or in-depth, and is somewhat one-sided.

### 4.4 Impacts on gut microbiota composition and metabolism

The fermentation process of plant polysaccharides exerts significant selective pressure on the gut microbiota. This not only directly influences polysaccharide metabolism but also substantially alters the composition and functionality of microbial communities. Gut bacteria can degrade plant polysaccharides that the host cannot directly utilize, producing beneficial metabolites such as SCFAs. Through this process, they restructure their own microbial communities, thereby mediating various physiological functions of polysaccharides.

#### 4.4.1 Reshaping microbial community composition

Plant polysaccharides function as prebiotic agents by reshaping metabolic niches rather than simply promoting predefined “beneficial” groups. Their fermentation drives competitive exclusion of less-adapted microbes and supports keystone degraders, initiating trophic cascades through cross-feeding interactions. For instance, *Bacteroides* cellulosilyticus degrades plant cell wall polysaccharides via secreted glycoside hydrolases (e.g., GH30 and GH157 families), releasing oligosaccharides such as β-1,3-galactose derivatives that remain incompletely utilized by the primary degrader but serve as substrates for secondary fermenters like *Bifidobacterium* breve UCC2003, which metabolizes them through its bga gene cluster, enabling cross-species carbon resource sharing (Fernandez-Julia et al., 2023). Genera frequently involved in such collaborative networks include *Bifidobacterium*, *Lactobacillus*, *Bacteroides*, and butyrate-producing members of the Firmicutes, such as *Faecalibacterium*, *Roseburia*, *Eubacterium*, and *Anaerostipes* (Li et al., 2016; McKee et al., 2021; Luo et al., 2022; Wu et al., 2022b; Zhang et al., 2024a). In another example of metabolic complementarity, *Bifidobacterium* adolescentis degrades starch or fructooligosaccharides to produce lactate and acetate, which are subsequently utilized by butyrate-producing bacteria like Eubacterium hallii or Anaerostipes caccae to generate butyrate via the acetyl-CoA pathway (Belenguer et al., 2006). In some cases, this reorganization of microbial energy flow may also coincide with a reduction in potentially harmful taxa, reflecting shifts in community stability and functional output rather than isolated compositional changes (Luo et al., 2022; Chen et al., 2024b).

**FIGURE 4:**
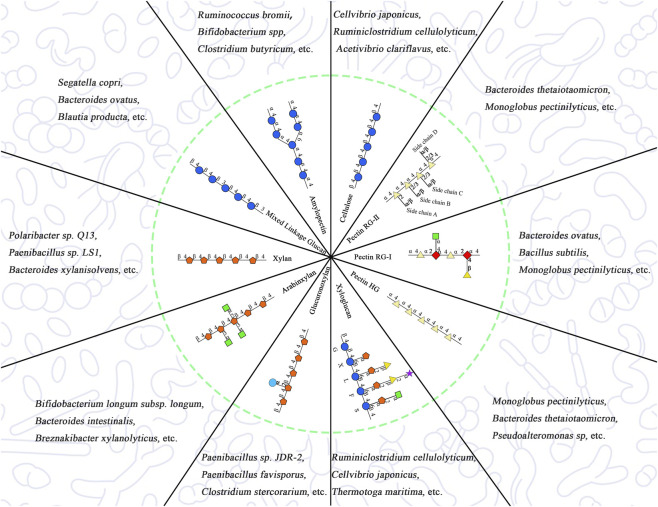
Schematic diagram of selective enrichment of degrading microbiota by structural characteristics of typical plant polysaccharides.

The ecological outcome of polysaccharide intervention is structure-dependent. Variations in monosaccharide composition, glycosidic linkage type, branching pattern, and molecular weight determine which microbes possess the requisite enzymatic machinery (e.g., PULs) to access a given substrate, thereby defining niche availability and network connectivity (Wu et al., 2021b) (Figure 4). For instance, β-glucans may preferentially engage butyrate-producing taxa within a cross-feeding network (Hu et al., 2024), whereas inulin tends to support keystone degraders such as *Bifidobacterium* that facilitate downstream metabolic interactions (Luo et al., 2022). Studies with polysaccharides from lotus leaf (LLP), okra pectin (OPP), Tremella (TFP), Sargassum (ST-P2), caravan (CCPP), Reishi, Astragalus (APS), and Polygonatum (PsPs) illustrate that such structural traits influence microbiota reorganization beyond taxonomic shifts, affecting ecosystem robustness and resilience during *in vitro* fermentation (Li et al., 2016; Fu et al., 2018; Yao et al., 2020; McKee et al., 2021; Wu et al., 2021b; Luo et al., 2022; Wu et al., 2022b; Zhang et al., 2024a). Collectively, these findings frame selective enrichment as part of broader ecological restructuring, emphasizing metabolic interdependence and community stability over binary classifications of microbial change. However, most existing studies ignore inter-individual variability. Differences such as baseline microbiota composition can lead to individual heterogeneity in the prebiotic effects of the same polysaccharide, limiting the development of personalized nutrition and precision prebiotics. In the future, it is necessary to combine individual flora background to construct multi-dimensional prediction models, promoting the precise transformation of the prebiotic effects of plant polysaccharides.

#### 4.4.2 Generating key metabolites

**FIGURE 5:**
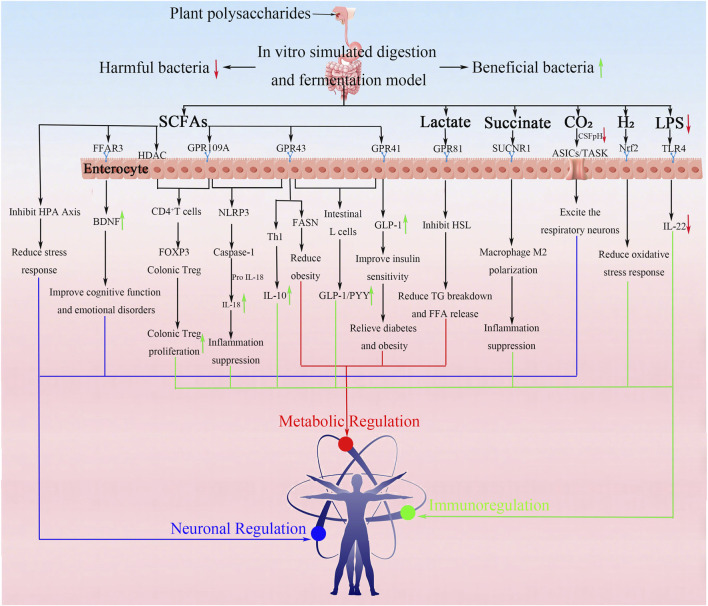
The metabolites of *in vitro* fermentation of plant polysaccharides and their possible route of action on body metabolism, immunity and neuroregulation.

The metabolic output of polysaccharide fermentation is the primary link between dietary fiber, gut microbiota, and host physiology (Figure 5).

SCFAs: Undigestible plant polysaccharides are fermented by gut microbiota in the colon to produce SCFAs, primarily consisting of acetic acid, propionic acid, and butyric acid (Figure 5). These SCFAs serve as key metabolic products with vital physiological functions. Butyric acid acts as the preferred energy source for colonic epithelial cells, propionic acid influences hepatic glucose and lipid metabolism, while acetic acid functions as a systemic energy substrate and precursor for lipid synthesis. All three SCFAs function through G protein-coupled receptors (GPRs) such as GPR41, GPR43, and GPR109A as signaling molecules, regulating immune and metabolic processes (van der Hee and Wells, 2021; Yan et al., 2024; Zhang et al., 2024a). Notably, the relative proportions of these SCFAs are highly dependent on the structural characteristics of the polysaccharides and the composition of active microbial communities. For relatively simple polysaccharides like β-glucan (e.g., PSP), they can significantly increase butyric acid production (Gao et al., 2024; Hu et al., 2024). Neutral polysaccharides such as arabic galactan (e.g., LBPS) are preferentially metabolized by *Bifidobacterium* into acetic and propionic acids (Ding et al., 2019). Conversely, complex polysaccharides like pectin (e.g., JHP, OPP) often generate higher concentrations of acetic acid during fermentation (Wu et al., 2021b; Wu et al., 2021c).

Other Metabolites: Besides SCFAs, fermentation generates other important molecules:

Succinate: Certain bacteria (e.g., *Bacteroides*) can produce substances that act on the SUCNR1 receptor, potentially regulating immune responses and colitis (Wu et al., 2022a). When barley β-glucan is metabolized by gut microbiota, it promotes *Bacteroides* proliferation and generates succinic acid (Mio et al., 2024).

Lactate: Produced by lactic acid bacteria (such as *Lactobacillus* lactis stimulated by tea polysaccharides (Tan et al., 2023)), these metabolites can lower intestinal pH and serve as cross-feeding substrates to generate propionic or butyric acids. Tan’s research demonstrates that the rhamnogalactosylated polysaccharide in tea polysaccharides (TP) effectively promotes the proliferation of *Lactobacillus*, thereby producing lactic acid.

Gases: The gut microbiota ferments indigestible plant polysaccharides, not only producing SCFAs but also generating gaseous metabolites such as hydrogen (H_2_), carbon dioxide (CO_2_), and methane (CH_4_). These gases are not merely byproducts of fermentation but play vital physiological roles. For instance, Coccus coccinea in the rumen can ferment the mannose side chains of Chitosan-Containing Plant Polysaccharide (CCPP) to produce H_2_, which exerts antioxidant effects through the Nrf2 pathway (Yao et al., 2020). Methane (CH_4_) and hydrogen sulfide (H_2_S) can also be produced by specific archaea and bacteria, respectively, with diverse physiological effects.

Potentially Detrimental Products: While primarily beneficial, fermentation under certain conditions or imbalances might contribute to products like lipopolysaccharide (LPS) associated with inflammation, although some polysaccharides like inulin can inhibit LPS effects (Yang et al., 2019).

In conclusion, the fermentation of plant polysaccharides is a dynamic and complex process involving intricate enzymatic deconstruction by the microbiota, leading to significant changes in polysaccharide properties. This process reciprocally shapes the microbial community structure and generates a diverse profile of metabolites, primarily SCFAs, which are central to mediating the health effects discussed in the following section. The specificity observed in these interactions underscores the importance of polysaccharide structure in determining fermentation outcomes.

## 5 Positive health effects driven by plant Polysaccharide fermentation and gut microbiota interactions

The positive health impacts of plant polysaccharides are not typically derived from the polysaccharides themselves but are overwhelmingly mediated by the metabolic activities of the gut microbiota during fermentation. As natural prebiotics, these polysaccharides fuel microbial communities, promoting the proliferation of beneficial bacteria (e.g., *Bifidobacterium*, *Lactobacillus*, butyrate producers) while potentially inhibiting harmful ones (Li et al., 2021; Yan et al., 2024; Zhang et al., 2024b). However, the core mechanism translating this microbial modulation into host benefits lies in the production and subsequent action of fermentation metabolites (Figure 5). While SCFAs (acetate, propionate, and butyrate) are the most studied and arguably most impactful mediators, other fermentation products like succinate, lactate, gases (H_2_), modified bile acids, and microbial metabolites of amino acids (e.g., tryptophan derivatives) also contribute to the complex physiological outcomes (van der Hee and Wells, 2021; Zhang et al., 2022b). These metabolites act locally within the gut and systemically after absorption, influencing metabolic regulation, immune function, neurological processes and even cardiovascular system through intricate host-microbe signaling networks, ultimately promoting homeostasis and disease prevention. Understanding the specificity of these fermentation-driven interactions offers promising prospects for developing targeted nutritional strategies (including personalized regimens) to maintain and enhance human health (Table 3).

**TABLE 3:** Summary Table of pharmacological studies on plant polysaccharides.

Name of polysaccharides	Source	Research model	Experimental duration	Control type	Tested dose	Pharmacological activities and Indexes	References
PKPS	*Polygonatum kingianum* Coll. et Hemsl	*In vitro* senescent cell model	48 h	Positive control group	0.5 mg/mL	Anti-aging; enhance proliferation activity, reduce β-galactosidase activity, and inhibit the level of SASP	Yang et al. (2023)
PKP1	*Polygonatum kingianum*	*In vitro* macrophage model	24 h	Positive control group	0.2 mg/mL	Immunomodulation; reduce the levels of inflammatory factors NO, IL-6 and TNF-α in LPS-induced macrophages	Han et al. (2025)
GLP	*Glycyrrhiza*	*In vitro* antioxidant model	84 h	Comparison before and after fermentation	20 μg/mL	Antioxidant; enhance the DPPH/hydroxyl radical scavenging capacity and reducing power	Du et al. (2025)
NCVP	*Nostoc commune* Vauch	*In vitro* antioxidant model	48 h	Comparison before and after fermentation	1 mg/mL	Antioxidant; enhance ABTS and hydroxyl radical scavenging capacity	Li et al. (2022c)
DOPS	*Dendrobium officinale*	Rat model of metabolic hypertension	7 weeks	Positive control group	200 mg/kg	Lower blood pressure and improve intestinal barrier function; increase acetic acid and upregulate GPR41/43	Li et al. (2022a)
PSVP	*Sanghuangporus vaninii*	Immunosuppressed mouse model	4 weeks	Positive control group	200 mg/kg	Immunomodulation; increase the levels of IgM and IgG	Yin et al. (2022)
OPP	*Abelmoschus esculentus*	*In vitro* digestion and fermentation model	48 h	Comparison before and after fermentation	20 mg/mL	Intestinal protection; produce propionic acid and improve insulin sensitivity	Wu et al. (2021b)
PSP	*Polygonatum spp*	*In vitro* antioxidant model	48 h	Comparison before and after fermentation	2.5 mg/mL	Antioxidant; scavenge DPPH free radicals and superoxide anions	Qi et al. (2024)
PE	*Ephedrae Herba*	Asthma rat model	21 days	Positive control group	137.71 mg/kg	Immunomodulation; regulate the immune imbalance of Th1/Th2 cells	Zhang et al. (2022a)
JPS	*Ziziphus Jujuba* cv. *Pozao*	*In vitro* macrophage model	24 h	Comparison before and after fermentation	200 mg/kg	Immunomodulation; upregulate IL-2, IL-4, IL-10, IFN-γ and TNF-α	Han et al. (2022)
LBP	*Lycium barbarum*	Mouse model of retinal ischemia	7 days	Positive control group	10 mg/kg	Neuroprotection; reduce neuronal death and glial cell activation	Yang et al. (2017)
CYPs	*Dioscorea opposita* Thunb	*In vitro* macrophage model	48 h	Comparison before and after fermentation	0.3–5 mg/kg	Immunomodulation; regulate TNF-α/IL-1β/IL-10/TGF-β	Yang et al. (2025b)
APs	*Aloe*	Mouse model of cognitive and behavioral disorders	24 weeks	Positive control group	200 mg/kg	Improve cognitive function; regulate gut-brain axis, reverse HFD-induced memory impairment and enhance spatial memory	Mo et al. (2024)
PSP	*Polygonum sibiricum*	Depression mouse model	7 days	Positive control group	200 mg/kg	Antidepressant; ameliorate HPA axis dysfunction and upregulate the levels of CORT, 5-HT and NE	Zhang et al. (2023)
DGPN	*Panax ginseng* C. A. Meyer	*In vitro* macrophage model	24 h	Positive control group	100 μg/mL	Immunomodulation; activate the TLR4/Myd88/NF-κB signaling pathway and promote the secretion of IL-6, TNF-α and NO	Xu et al. (2025a)

### 5.1 Metabolic regulation via fermentation metabolites

Plant polysaccharides, after microbial fermentation, can significantly affect the host’s glycolipid metabolism.

Glycogenolysis: The SCFAs produced by polysaccharide fermentation significantly influence glucose and lipid metabolism. This regulation occurs through enhancing insulin sensitivity and glucose homeostasis, inhibiting fat synthesis, promoting lipid oxidation, reducing adipose tissue inflammation, and modulating bile acid metabolism to optimize lipid processing. Propionic acid, a crucial substrate for hepatic gluconeogenesis, is converted into glucose in the liver to provide energy. For instance, okra pectin polysaccharide (OPP) generates propionic acid through fermentation, and this propionic acid improves insulin sensitivity and helps prevent obesity and type 2 diabetes by regulating hepatic gluconeogenesis (Wu et al., 2021b). Fermented astragalus polysaccharide increases *Bacteroides* abundance and butyrate levels, effectively addressing glucose metabolic disorders in type 2 diabetes models (Zhang et al., 2024a). Similarly, processed Polygonatum polysaccharides produce butyrate (Luo et al., 2022; Zhang et al., 2024b). Studies demonstrate that butyrate improves insulin resistance, promoting long-term metabolic stability (Tang et al., 2022; Chen et al., 2024c). Notably, fermented platycodon polysaccharide (PS) stimulates propionic acid production and activates intestinal GPR43 receptors, inhibiting fatty acid synthase (FASN) expression to reduce obesity in mouse models (Ke et al., 2023). Furthermore, propionic acid has been shown to decrease hepatic fat accumulation in obesity models (Wang et al., 2019b).

### 5.2 Immune modulation orchestrated by fermentation

Polysaccharides can be fermented by intestinal flora, and can synergistically enhance the immune function of the body through multiple ways by regulating local and systemic immunity (including natural immunity and acquired immunity).

#### 5.2.1 Supporting role of intestinal barrier function in immune regulation

The integrity of the intestinal barrier serves as a critical basis for the realization of this immunomodulatory function. The enhanced protective effects of polysaccharides on the intestinal barrier during fermentation involve three layers: mechanical (epithelial cells and tight junction proteins), chemical (mucosal layer), and biological (gut microbiota). Fava polysaccharide, after fermentation, reduces intestinal permeability by upregulating tight junction proteins like Occludin and ZO-1, thereby improving the mechanical barrier function (Ye et al., 2024). Wang’s study demonstrated that in colitis mouse models, plant polysaccharides reaching the colon via the digestive tract promote goblet cell secretion of mucin (MUC2), thickening the protective mucus layer to enhance chemical barrier function (Wang et al., 2024c). Fermentation of Polygonatum polysaccharide (PSP) enriches Roscheia species producing butyrate, strengthening the biological barrier function (Hu et al., 2024). Studies indicate butyrate improves intestinal mucosal integrity, thereby enhancing chemical barrier performance (Topping and Clifton, 2001; den Besten et al., 2013). Fermented barley β-glucan generates succinic acid, which enhances intestinal barrier function (Mio et al., 2024). This may be because succinic acid, as an intermediate product of the tricarboxylic acid cycle (TCA cycle), directly enters mitochondria for energy metabolism, providing ATP to intestinal cells and supporting mucosal renewal and repair (Mills and O’Neill, 2014).

#### 5.2.2 Regulation of innate immunity by the fermentation process

The fermentation process can influence the regulation of polysaccharides on natural immunity: After fermentation, plant polysaccharides regulate the body’s innate immune response by mediating immune cell functions and cytokine secretion. Polygonatum polysaccharide (PKP1) fermentation increases the production of probiotics (such as Bifidobacteria) and SCFAs, while significantly reducing NO, IL-6, and TNF-α levels in LPS-induced RAW264.7 macrophages, thereby affecting natural immune function (Han et al., 2025). *Dioscorea opposita* polysaccharides (CYPs) exhibit enhanced immunomodulatory activity after fermentation, regulating tumor necrosis factor-α, interleukin-1β, interleukin-10, and transforming growth factor-β levels in RAW264.7 macrophages (Yang et al., 2025b). Legume seed coat polysaccharides (LSCPs) produce butyrate through fermentation, which inhibits pro-inflammatory cytokines IL-6 and TNF-α release via the TLR4/MyD88 pathway, thereby modulating natural immune responses (Ding et al., 2025). In addition, relevant studies have shown that butyrate can alleviate inflammatory responses by inhibiting the NF-κB pathway of the innate immune response in macrophages (Chen et al., 2024c). Fermented Phyllanthus leaves polysaccharide (PM) significantly promotes macrophage proliferation, phagocytosis, and secretion of IL-6, IL-1β, and TNF-α, demonstrating remarkable immunomodulatory capabilities (Chen et al., 2025).

#### 5.2.3 Regulation of adaptive immunity by the fermentation process

The fermentation process can influence the regulation of polysaccharides on acquired immunity: After fermentation, plant polysaccharides regulate the body’s acquired immune response by mediating T lymphocytes and B lymphocytes functions and immunoglobulin (Ig) secretion. Barbary Wolfberry polysaccharide (LBPS) fermentation produces acetic acid and propionic acid, which upregulate FOXP3+ regulatory T cells and can alleviate autoimmune diseases by inhibiting histone deacetylase HDAC (Ding et al., 2019). Mulberry flavonoid polysaccharide (PSVP) fermentation increases the content of immunoglobulins IgM and IgG, thereby regulating the body’s acquired immune response (Yin et al., 2022). In asthma models, it was found that fermented Ephedra polysaccharide can reduce Th2-type bias, lower IL-4 levels, and improve acquired immune response (Zhang et al., 2022a). Fermented Panax notoginseng polysaccharide can enhance the body’s acquired immune response by inhibiting excessive inflammatory responses of Th17 cells (Raposo et al., 2024).

### 5.3 Neuromodulation through the gut-brain axis: A fermentation link

Polysaccharides deeply affect the function of the gut-brain axis through the interaction with intestinal microorganisms, thus closely linking the activity of intestinal flora with brain nerve function (Khan et al., 2024).

Intestinal microorganisms ferment plant polysaccharides to produce SCFAs, which can cross the blood-brain barrier (BBB) and directly affect microglia and neurons. We found that after fermentation, barbary wolfberry polysaccharides can exert neuroprotective functions, accompanied by increased SCFAs and improved gut microbiota (Feng et al., 2024). This neuroprotective effect is likely to be caused by butyrate. Studies have shown that butyrate can directly cross the BBB, influence microglia function, promote the expression of brain-derived neurotrophic factor (BDNF), and may affect cognitive function and mood through activation of FFRAR3 receptors (a GPR) (Ge et al., 2023). Furthermore, the fermentation of plant polysaccharides can also affect the levels of metabolites associated with neuroactivity. For example, gut microbiota can metabolize tryptophan (Trp), while certain plant polysaccharides may indirectly increase serotonin (5-HT) levels in the colon by promoting *Lactobacillus* proliferation and its Trp metabolism, ultimately influencing neural function (Ye et al., 2024). For instance, the fermentation of Polygonatum polysaccharide (APS) increased the abundance of *Bifidobacterium*, thereby elevating tryptophan and 5-hydroxytryptophan levels (p < 0.05). Such changes in neuroactive metabolite levels significantly impact the body’s neural function (Mi et al., 2025); similarly, fermented Barbary wolfberry polysaccharides can regulate glutamate metabolism, thereby exerting indirect neuroprotective effects (Yang et al., 2017).

After fermentation, plant polysaccharides can regulate the nervous system through the HPA axis. The hydrolysis products (SCFAs) and other microbial metabolites exert neuroregulatory effects by modulating the hypothalamic-pituitary-adrenal (HPA) axis (a key stress response system) (Zhao et al., 2023). Studies have shown that fermented aloe polysaccharides can inhibit excessive activation of the HPA axis, thereby reducing stress responses in mice. This effect is closely associated with elevated propionic acid levels generated during fermentation (Mo et al., 2024). After fermentation, Polygonatum sibiricum polysaccharides alleviate depression-like behaviors by reducing the level of stress hormones (corticosterone). The decreased corticosterone level weakens its activation of the HPA axis, thereby exerting a neuroprotective effect (Zhang et al., 2023).

### 5.4 Effects on the cardiovascular system

#### 5.4.1 Blood pressure regulation

The fermentation of plant polysaccharides by intestinal flora can significantly affect blood pressure homeostasis, which is mainly related to the active metabolites produced in this process, such as short-chain fatty acids (SCFAs), lactic acid and succinic acid.

SCFAs can regulate blood pressure by activating G protein-coupled receptors (GPR41/43). Yang’s study found that GPR41 deficiency reduces sympathetic nervous system activity, while mice with dual knockouts of GPR41/43 exhibit more severe hypertension and renal macrophage infiltration induced by angiotensin II (Yang et al., 2020). Li’s research demonstrated that Dipsosides (DOPS) from *Dendrobium officinale*, when fermented *in vivo*, lower blood pressure in metabolic hypertensive rats, improve gut microbiota, strengthen intestinal barriers, significantly increase acetic acid levels, and enhance GPR43/41 expression (Li et al., 2022a). These studies confirm that acetic acid inhibits renin release and induces vasodilation by binding to GPR41 in the kidneys and blood vessels. Intravenous administration of acetic acid reduced mean arterial pressure by 54 mmHg in mice and decreased heart rate by 233 bpm, indicating its direct effect on cardiac contractility (Poll et al., 2019). Bisaccharide-metabolized polysaccharides (WMFPs), when fermented by gut microbiota, can regulate blood pressure by inducing endothelium-dependent relaxation in rat mesenteric arteries (Wang et al., 2019a). Studies indicate that this blood pressure regulation may be mediated through butyrate, which enhances intestinal barrier function by inhibiting HDAC activity. This mechanism reduces endotoxin translocation and systemic inflammation, thereby improving vascular endothelial-dependent vasodilation (Robles-Vera et al., 2020; Yang et al., 2020). Additionally, a derivative of β-D-glucan (CMG), after digestion and fermentation in rats, exerts antihypertensive effects by modulating sympathetic tone to improve stress reflex sensitivity (Bezerra et al., 2021). Research demonstrates that SCFAs (butyrate) activate the vagal afferent fibers of the solitary nucleus (NTS), which inhibits excessive sympathetic nerve excitation and indirectly lowers blood pressure (Welathanthree et al., 2025). Lactic acid and succinic acid, as fermentation products of plant polysaccharides, also exert certain effects on blood pressure homeostasis. Fermented low-molecular-weight plantain polysaccharides can significantly promote the proliferation of *Lactobacillus*, thereby producing lactic acid (Hu et al., 2018). Studies indicate that lactic acid regulates blood pressure through two mechanisms: On one hand, probiotics like *Lactobacillus* ferment to produce lactic acid, which lowers intestinal pH and enhances calcium/magnesium ion absorption, indirectly improving vascular tone and thus lowering blood pressure. On the other hand, excessive lactic acid accumulation may activate the renin-angiotensin system (RAS) or induce metabolic acidosis, leading to elevated blood pressure (Robles-Vera et al., 2020; Keller et al., 2024). Fermented barley β-glucan not only improves gut microbiota composition but also promotes succinic acid production (Mio et al., 2024). Succinic acid, through binding to SUCNR1 receptors in the juxtaglomerular space, stimulates renin release and activates the RAS system, thereby raising blood pressure. High-salt diets increase intestinal succinic acid production, showing positive correlation with 24-h ambulatory blood pressure in hypertensive patients (Huart et al., 2019).

#### 5.4.2 Atherosclerosis (As)

After being fermented by intestinal flora, plant polysaccharides can play a certain protective role in atherosclerosis.

Clinical studies have revealed that As patients exhibit significantly reduced butyrate levels in their feces (Xu et al., 2025b). In animal models, supplementation with butyrate has been shown to reduce plaque area by 42% (Kasahara et al., 2018), suggesting that SCFAs (the primary metabolites of plant polysaccharide fermentation) may provide protective effects against As. Chen et al. demonstrated that fermented dietary fiber (pectin) promotes the proliferation of butyrate-producing bacteria and enhances butyrate production. This metabolite alleviates atherosclerosis in apolipoprotein E-deficient mice by inhibiting intestinal cholesterol absorption (Chen et al., 2018b). These findings indicate that both gut microbiota dysregulation and SCFA depletion may exacerbate As-related pathologies. Indeed, our research confirms that microbial rhythm disorders lead to SCFA reduction, which subsequently activates the cIAP1/2-mediated TNF/NF-κB pathway. This pathway intensifies endothelial damage and plaque instability in As patients, thereby worsening endothelial dysfunction (Liu et al., 2025a). Therefore, fermented plant polysaccharides can exert protective effects on As by regulating gut microbiota homeostasis and SCFA production.

## 6 Perspectives

Significant progress has been made in studies on the interaction between plant polysaccharides and gut microbiota, with fermentation being the central process. Current research reveals that these carbohydrates; anti-digestive properties enable their passage to the colon, where they undergo transformation through microbial enzymatic systems while influencing both polysaccharide structure and microbial ecology. However, despite these advancements, numerous research directions remain to be explored in order to fully unlock their health benefits.

While *in vitro* simulation models hold significant value, bridging the gap between these systems and real-world biological environments remains a persistent challenge. Current models often oversimplify the complex, dynamic, and spatially organized intestinal environment. Future research should leverage advanced biomimetic systems such as organoids and microfluidic “intestinal-on-a-chip” platforms (potentially incorporating host immune metabolites) to better replicate physiological conditions (Fletcher et al., 2025). Integrating these sophisticated *in vitro* tools with well-designed animal studies (particularly human clinical trials) is crucial for validating dose-response relationships, assessing safety and efficacy, and evaluating impacts on host physiological states (Kuldeep Vinchurkar et al., 2022).

Secondly, elucidating precise structural-function relationships is crucial. The remarkable diversity of plant polysaccharides requires a deep understanding of how specific structural motifs (such as glycosidic bond types, branching patterns, polymerization degrees, presence of uronic acids, or modifications like acetylation and sulfation) determine substrate recognition by microbial enzymes (particularly those in the polysaccharide utilization genes PULs), influence fermentation kinetics, shape enriched microbial communities, and regulate metabolite profiles (e.g., short-chain fatty acid ratios and succinic acid production). This necessitates the integration of high-throughput screening with detailed structural characterization and fermentation analysis.

Third, exploring microbial “dark matter” and metabolic complexity requires advanced technologies. Integrating multi-omics approaches including metagenomics (identifying microbial species and their functions), metatranscriptomics (analyzing gene expression patterns), metaproteomics (detecting enzyme activity levels), and metabolomics (monitoring metabolite production) is essential for unraveling the functional intricacies of polysaccharide fermentation ecosystems. These tools can help uncover novel degradation pathways, identify key microbial roles and synergistic interactions (cross-feeding networks), and discover previously unrecognized bioactive fermentation metabolites.

Fourth, dietary background and food matrix effects cannot be ignored. In actual diets, plant polysaccharides are often part of complex food matrices, and their digestive and fermentative effects are significantly influenced by processing methods (such as heat treatment and drying), co-ingested nutrients including proteins and fats, as well as polyphenolic metabolites. These factors can alter the structural characteristics of polysaccharides, their contact probability with gut microbiota, and the utilization of polysaccharides by gut microbiota. Therefore, future research should comprehensively consider the above factors to make the findings more consistent with the real physiological environment.

Fifth, individual differences are a key factor influencing the effectiveness of polysaccharide interventions. Host genetics, baseline gut microbiota composition, age, lifestyle, and medication use can all affect polysaccharide fermentation efficiency and metabolite production. Research focusing on understanding these individual differences is critical to moving toward personalized nutritional strategies, which tailor polysaccharide recommendations based on predicted fermentation capacity and health goals.

Finally, the knowledge gained from this research demonstrates significant translational potential. A deeper understanding of polysaccharide fermentation mechanisms could drive the development of next-generation highly specific and effective prebiotics, as well as novel synbiotic formulations. Postbiotics may also be engineered through purified fermentation metabolites or engineered microbial strains. This study provides guidance for optimizing food formulation strategies to enhance gut health benefits, while offering a foundation for novel therapeutic approaches targeting metabolic, immune, and neurological disorders associated with gut microbiota dysbiosis. Ongoing exploration of polysaccharide fermentation regulation mechanisms is poised to unlock innovative applications across food science, nutrition, and medical fields.

## 7 Conclusion

In this review, we systematically examine the digestive properties of plant polysaccharides and their complex interactions with gut microbiota driven by fermentation. A key finding is that the biological activity of these complex carbohydrates primarily stems not from direct host absorption, but rather from microbial fermentation in the colon. Their journey begins with remarkable resistance to digestion in the upper gastrointestinal tract, a critical feature that enables them to reach the site of intestinal microbiota colonization.

Upon entering the colon, dynamic interactions immediately commence. The microbiota actively dismantle polysaccharide structures using complex enzyme systems, including CAZymes embedded in the polydextrose utilization gene loci (PULs). This fermentation process significantly alters both the physicochemical properties (e.g., viscosity reduction and solubility enhancement) and structural characteristics (glycosidic bond cleavage, molecular weight reduction, and functional group modification) of polysaccharides. Simultaneously, the fermentation of specific polysaccharides exerts strong selective pressure by promoting beneficial microorganisms and influencing overall microbial diversity, thereby shaping the gut microbiota. Most crucially, the fermentation process generates a series of bioactive metabolites, with SCFAs being particularly pivotal. These metabolites act as key signaling molecules that mediate communication between gut microbiota and host cells, ultimately influencing energy metabolism, immune homeostasis, and gut-brain axis function.

Despite significant progress, further exploration through multi-omics technologies, advanced *in vitro* models, and human studies is essential to fully elucidate the structural specificity mechanisms of polysaccharide fermentation, understand inter-individual differences, and investigate dietary synergistic interactions. Ultimately, this deepened understanding of fermentation-driven interactions holds tremendous potential for developing targeted nutritional and therapeutic strategies aimed at preventing and managing chronic diseases, optimizing gut function, and promoting overall human health. The microbial fermentation potential of plant polysaccharides represents a promising frontier in nutritional science and preventive medicine.
